# Advancing our understanding of the neurobiology of anorexia nervosa: translation into treatment

**DOI:** 10.1186/s40337-017-0169-8

**Published:** 2017-12-01

**Authors:** Lasse Bang, Janet Treasure, Øyvind Rø, Andreas Joos

**Affiliations:** 1 Regional Department for Eating Disorders, Division of Mental Health and Addiction, Oslo University Hospital, P.O. Box 4956 Nydalen, 0424 Oslo, Norway; 2 Department of Psychological Medicine, King’s College London, Institute of Psychiatry, Psychology and Neuroscience (IoPPN), London, UK; 3 Division of Mental Health and Addiction, Institute of Clinical Medicine, University of Oslo, Oslo, Norway; 4 Department of Psychosomatic Medicine and Psychotherapy, Medical Center, Faculty of Medicine, University of Freiburg, Freiburg, Germany

**Keywords:** Eating disorders, Anorexia nervosa, Neurobiology, Treatment, Neuropsychiatry, Translation

## Abstract

A wealth of studies has investigated the neurobiological underpinnings of anorexia nervosa. In our letter to the editor, we point to a number of ways in which the advances in our understanding of the neurobiology of anorexia nervosa - focusing on neuroimaging studies of brain structure and function - can be translated into treatment. We point to how such advances can: inform psychological treatment, be implemented in psychoeducation, point to novel therapeutic targets, lead to the identification of biomarkers, and expand our vocabulary for how we think and talk about anorexia nervosa.

Anorexia nervosa (AN) is a severe mental disorder predominantly affecting young women. Less than half of patients recover following treatment. For the past 20 years, substantial research has been invested into the identification of the neurobiological underpinnings. Structural brain imaging studies have shown gray and white matter changes which generally normalize with recovery [1]. Functional brain imaging studies have demonstrated alterations in fronto-striato-limbic circuits, suggesting aberrant cognitive control, reward, and emotion processes [2]. This suggests AN psychopathology emerge as a result of altered interactions between top-down and bottom-up circuits [3], possibly related to core disturbances in how the brain processes salient stimuli [2]. Below we point to examples of how advances in our understanding of the neurobiology of anorexia nervosa, focusing on neuroimaging studies of brain structure and function, can be translated into treatment.

## Neurobiological studies can inform and influence psychological treatments

For example, the consistent findings of altered brain structure and function provide biological evidence that the brain is affected by prolonged malnutrition. This underscores the importance of early weight-rehabilitation. Awareness of the strong structural brain alterations in the acute state and their normalization following weight-gain can influence the *attitude of the therapist and carers*, and the ethos of services. Furthermore, functional brain alterations are present in both ill and recovered patients, and are associated with traits such as anxiousness [2]. This underscores the importance of working *with* as opposed to *against* these traits during treatment. Teaching patients to use their traits in a constructive as opposed to destructive manner may be valuable. A recent study showed that neural activations moderated the relationship between stress and binge-eating behavior in everyday life among patients with bulimia nervosa [4]. Such studies holds promise in identifying neural mechanisms underlying everyday pathological behaviors, and could prove helpful in the development of novel treatments. Furthermore, neurobiological models can be integrated into treatment as *psychoeducation* for patients and their families as described in multimodal interventions targeting both patients and carers [5]. From a psychodynamic perspective, neurobiological models can be regarded as “third objects” which might decrease or circumvent resistance to therapy and change. Such psychoeducation may increase patients’ understanding of their own disorder, and boost motivation to overcome their anxieties and gain weight. One study reported that neurobiological-centered psychoeducation had beneficial effects on patients and their supports [5].

## Identification of affected brain circuits may point to valuable therapeutic targets

Neuromodulatory treatments for AN under investigation include deep brain stimulation and transcranial magnetic stimulation. Deep brain stimulation of the subcallosal cingulate shows promising effects on weight, mood, and anxiety in treatment-resistant patients at 1-year follow-up [6]. Transcranial magnetic stimulation may also have therapeutic potential [7], although larger studies are needed. These treatments may augment traditional forms of treatment by producing changes in core symptoms or comorbidities, particularly in severely affected treatment-resistant patients. Striato-limbic circuits are involved in non-conscious and automatic processes related to reward and emotion, which are of interest as targets of health-related behavior change [8]. Targeting these subcortical circuits could be accomplished with established psychological interventions such as exposure treatment and inhibition training.

## Neurobiological research can identify biomarkers

These can be used in the evaluation and formulation of diagnosis, prognosis, and treatment planning. For instance, brain characteristics could be used to predict clinical course or outcome. One study reported associations between neural activation at admission and 1-year outcome in AN [9]. A recent investigation also showed that structural brain characteristics could be used to correctly classify AN cases and controls with a 74% accuracy [10]. If such tools are realized, they will enable treatment individualization, e.g. by suggesting treatment pathways for a given patient.

## Neurobiological studies improve our understanding of the pathophysiology

Understanding the mechanisms that underlie the development and maintenance of AN may form the basis for developing new therapeutic strategies. However, it will also expand our vocabulary for how we think and talk about AN, which will be beneficial for patients, carers, and clinicians. The enriched language may reduce stigma: Unlike many psychological terms (e.g. rigid thinking) there are no negative connotations associated with neurobiological terms such as “hyperactive amygdala”. Moreover, a better neurobiological understanding can improve awareness and encourage early detection and treatment for eating disorders. This enhanced knowledge and attitude towards the disease will also have an impact in the teaching of future therapists.

To conclude, understanding the neurobiology of AN holds a variety of treatment implications, and we have highlighted some of these in our letter. New brain-based treatments are of interest but there are wider implications in relation to psychological treatments, and in terms of understanding, awareness, and stigma. Neurobiological research findings should complement and be integrated with psychological and family-based approaches – and not be a substitute. Such a complementary approach reflects true biopsychosocial understanding, which is the core of psychosomatic medicine.

## References

[1] Bernardoni F, King JA, Geisler D, Stein E, Jaite C, Nätsch D (2016). Weight restoration therapy rapidly reverses cortical thinning in anorexia nervosa: a longitudinal study. NeuroImage.

[2] Treasure J, Zipfel S, Micali N, Wade T, Stice E, Claudino A (2015). Anorexia nervosa. Nat Rev Dis Primers.

[3] Schmidt U, Campbell IC (2013). Treatment of eating disorders can not remain ‘brainless’: the case for brain-directed treatments. Eur Eat Disord Rev.

[4] Fischer S, Breithaupt L, Wonderlich J, Westwater ML, Crosby RD, Engel SG (2017). Impact of the neural correlates of stress and cue reactivity on stress related binge eating in the natural environment. J Psychiatr Res.

[5] Hill L, Peck SK, Wierenga CE, Kaye WH (2016). Applying neurobiology to the treatment of adults with anorexia nervosa. J Eating Dis.

[6] Lipsman N, Lam E, Volpini M, Sutandar K, Twose R, Giacobbe P (2017). Deep brain stimulation of the subcallosal cingulate for treatment-refractory anorexia nervosa: 1 year follow-up of an open-label trial. The Lancet Psychiatry.

[7] McClelland J, Kekic M, Campbell IC, Schmidt U (2016). Repetitive transcranial magnetic stimulation (rTMS) treatment in enduring anorexia nervosa: a case series. Eur Eat Disord Rev.

[8] Hollands GJ, Marteau TM, Fletcher PC (2016). Non-conscious processes in changing health-related behaviour: a conceptual analysis and framework. Health Psychol Rev.

[9] Schulte-Rüther M, Mainz V, Fink GR, Herpertz-Dahlmann B, Konrad K (2012). Theory of mind and the brain in anorexia nervosa: relation to treatment outcome. J Am Acad Child Adolesc Psychiatry.

[10] Soares J, Lavagnino L, Mwangi B, Cao B, Shott ME, Frank G. 596. Cortical thickness patterns in anorexia nervosa: individualized prediction model and correlations with key symptoms. Biol Psychiatry. 2017; 81 (10, Supplement):S241.

